# Supporting Educational Excellence in Diversity (SEED): faculty development and allyship

**DOI:** 10.1186/s12909-024-06403-0

**Published:** 2025-02-20

**Authors:** Puja Chadha, Esther H. Kang, Victoria Ngo, Rebecca Jorrin, Khoban Kochai, Kupiri Ackerman-Barger, Hendry Ton

**Affiliations:** 1 Office of Health Equity, Diversity, and Inclusion, University of California Davis Health, Sacramento, CA USA; 2 Department of Psychiatry and Behavioral Science, University of California Davis School of Medicine, Sacramento, CA USA; 3 Medicine-Pediatrics Residency Program, University of California Los Angeles Health, Los Angeles, CA USA; 4 Betty Irene Moore School of Nursing, University of Davis Health, Sacramento, CA USA

**Keywords:** Faculty development, Communication skills, Curriculum development/evaluation, Professional development, Continuing medical education, Cultural Humility, Emotional intelligence

## Abstract

**Introduction:**

Bias, privilege, microaggressions, and other forms of mistreatment negatively impact students’ learning, professional development, wellness, and identity. Supporting Educational Excellence in Diversity (SEED) is an innovative faculty development curriculum co-founded and co-designed by medical students/trainees. SEED is unique given it provides vital yet practical communication tools and strategies to support cultural humility when navigating critical conversations related to diversity, inclusion, and harm.

**Objective:**

This curriculum helps faculty to identify, redress, and prevent mistreatment within clinical and non-clinical learning environments while cultivating cultural humility.

**Methods:**

SEED incorporated a sequential hybrid model approach through the use of interactive online modules, virtual asynchronous self-reflection assignments, followed by in-person discussions with role-play opportunities. Using novel tools, it taught faculty core knowledge and strategies to facilitate discussions in navigating harms related to diversity and inclusion. Authors measured impact via self-reported, de-identified pre-and-post questionnaires.

**Results:**

An idependent t-test analysis pre-post-test study of 67 faculty participants revealed statistically significant (*P* < .001) differences from pre to post on all items with one item statistically significant at a *p* < .05. The overall effect size was 1.65 showing significant improvement in participants’ self-perceived ability to identify and address microaggressions, privilege, sources of bias, and related harm in the clinical and learning environments. These improvements were identified within themselves, faculty peers, teaching curricula, and teaching modalities. SEED was adopted health system-wide through customized departmental faculty offerings given the statistically and practically significant change in learning, awareness, and attitudes for respondents. SEED is currently under consideration as a maintenance of certification self-assessment course by a national board.

**Conclusion:**

The innovative SEED faculty development curriculum was co-created and designed by students/trainees. SEED shows promising results in improving participants’ foundational learning in promoting inclusive change to enhance learning environments for healthcare trainees. SEED is unique in providing vital yet practical communication tools and strategies to support cultural humility when navigating critical conversations related to diversity, inclusion, and harm.

## Introduction

The academic climate strongly influences students’ learning, professional development, wellness, and identity. Medical student mistreatment, particularly as it relates to race, ethnicity, gender, and sexual orientation continues to be a significant problem across all medical schools [1, 2]. It negatively impacting student experience, learning environment, and educational outcomes [3]. Additional data is also emerging on the negative impact of mistreatment in the learning environment, particularly when related to gender, sexual orientation, and race/ethnicity. [4] Prior studies have shown that medical students who identify as female, Asian, LGBTQ+, or underrepresented minorities bear the undue burden of mistreatment throughout their training [4]. Kumagai et al. [5] and Hill et al. [4], emphasized a need to focus on students’ and healthcare providers’ attitudes as they related to culturally responsive patient care—that begins with modeling how we care for students [4]. Literature also suggests a lack of consensus on how cultural competence should be taught in medical school.” [6–8] As competency refers to the ability to comprehend and understand, the term, “cultural competency” may imply a false sense of mastery of another’s culture. Limited resources are available to support individuals on developing self-awareness and the skills to navigate these critical conversations. To address the unintended consequences of a narrow or limited understanding of cultural competence, Tervalon and Murray-García (1998) proposed a critical shift to *cultural humility* [8] and later Curtis et al. (2019) suggested a move to *cultural safety.* [9]

Cultural Humility is defined as, “a lifelong process of self-reflection and self-critique, whereby the individual not only learns about another’s culture, but one starts with an examination of [their] own beliefs and cultural identities.” [8] Cultural safety builds upon cultural humility and in particular, expands on the notion of power differentials [8, 9]. A key difference between the concepts of cultural competency and cultural safety is the notion of ‘power’. Cultural safety foregrounds power differentials within society, it requires health professionals to reflect on interpersonal power differences (their own and that of the trainees or patients), and how the transfer of power within multiple contexts can facilitate appropriate care for underrepresenting individuals and arguably for all trainees and patients [9]. Cultural safety [9] with cultural humility [8] is critical to achieving health equity [10]. Training that outlines these essential principles with practical steps to operationalize this approach in healthcare organizations and workforce development is important.

Systematic reviews of cultural competence-related trainings have shown to be effective in improving the knowledge of health professionals and suggests that it improves attitudes, skills, and behaviors with demonstrated beneficial outcomes [5, 11, 12]. Faculty development that is safe and interactive promotes an effective and safe growth mindset [13]. Campbell et al. [14] highlights the use of trusted peer contributors rather than outside experts to foster more collaborative positive relationships. A collaborative relationship promotes a growth mindset, thus leading to a more lasting rewarding experience. These safe experiences avoid the pitfalls of expert evaluation, which can feel punitive and reinforce the fixed mindset. The flipped classroom inverts traditional teaching methods, delivering lecture instruction outside class, and devoting class time to problem-solving; the teacher’s role becomes that of a learning coach and facilitator [15]. In-class sessions can then be transformed into safe spaces for dialogue, skills practice, empowerment, and the cultivation of one’s humanistic identity [16] as an educator.

Supporting Educational Excellence in Diversity (SEED) Faculty Development Curriculum was created through a collaborative effort of students, staff, and faculty to address student-driven concerns related to microaggressions, bias, and privilege in medical school education. The course aims include harnessing and enhancing cultural mindfulness and awareness during interactions to empower faculty as agents of change and lead in negotiating issues when unintentional harmful outcomes occur due to a lack of diversity and inclusion [17, 18]. SEED is unique as it provides vital yet practical communication tools and strategies to support cultural humility when navigating critical conversations related to diversity, inclusion, and harm. The inclusion of the concept of “harm” may possibly support healthcare faculty buy-in to the critical need of better addressing areas related to diversity, equity, and inclusion. This article describes the faculty development initiative and the creation of a curriculum that promotes faculty members’ capacity to cultivate safer learning spaces for students and trainees. A separate, future article will investigate the clinical impact of this initiative.

## Methods

### Characteristics and strategies of SEED

The SEED Curriculum aimed to improve the trainee learning environment, a strong influencer of learning, professional development, wellness, and identity of trainees. Another aim of the SEED Curriculum was to equip educators with knowledge and tools to improve relationships between healthcare professionals and students in both clinical and non-clinical educational settings.

The curriculum introduced elements of an inclusive healthcare learning environment and their relationship to teaching excellence; highlighted mistreatment data in the educational and clinical environments; examined medicine as a culture; reviewed how mistreatment occurs and how to address them; and reviewed topics of bias, privilege, microaggressions, and stereotype threat, as well as how these factors impact educational outcomes and the learning environment. It was designed to bring attention to the student experience in healthcare education, unintentional harm, and navigating conflict with components of healing relationships after harm occurs. It highlighted how learning can also help in clinical care, faculty peer interactions, and with other colleagues in treatment teams.

### Learning objectives

Learning objectives focus on enhancing skills of perspective-taking; application of tools from the course to navigate conflict and mistreatment; providing effective feedback to trainees and peers; developing effective allyship, as well as effective apologizing. Overarching goals are to enhance emotional intelligence and mindfulness in professionalism; to enhance giving feedback, and to explore joint problem-solving while empowering allyship. We explored power differentials relating to structural inequity within healthcare systems through case examples and discussion. We also integrated how their own power as faculty versus lack of power for trainees impact experience and perpetuate inequities. Overall, the training aims to help each participant to more effectively support inclusion while addressing harm as agents of change.

### Course structure

The curriculum was designed using a flipped-classroom model in which participants completed interactive online modules with reflection assignments covering core information before an in-class interactive session where the learning content would be applied. The flipped classroom modality was selected as it has been shown to have a higher yield in healthcare education than traditional modalities [19]. Online modules and self-reflection assignments provided data, explained current issues, and presented core strategies in the context of their application. The curricular design was also informed by data from the Association of American Medical Colleges (AAMC) Graduation Questionnaire [20], results of an in-house survey that assessed faculty self-perceived needs for cultural humility training, and aggregates of real-life cases in which medical students experienced cultural harm. Cultural harm refers to the harm experienced toward a community from a dominant group [21]. The online interactive modules and self-reflection assignments were designed to empower self-awareness, with the aim for each participant to become an agent of change on issues including microaggressions, healthcare and education disparities, privilege, race, ethnicity, gender, and sexual orientation. Completing the online components was key preparation for participants to engage in interactive in-person sessions. The in-class session objectives focused on team-building, role-playing, and interactive discussion to facilitate strengthened allyship [22] for faculty educators. For the in-class session, we offered a single, 4-hour session or two 2-hour session depending on department availability. The in-class session allowed for group discussion, self-reflection and reviewing of cases with role play. Discussion and peer role play are effective modalities to learn and enhance communication skills [23]. 

### Curriculum overview

The four core segments of SEED are: (1) What We Know Now, (2) Self-reflection & Management, (3) Inclusive Communication, (4) Diving Deeper with Consolidation.

1. *What We Know Now*: Defines essential core terms, and provides relevance of the training utilizing AAMC Medical Student Graduation Questionnaire data on mistreatment, institution-specific results of a faculty needs assessment on cultural training, and actual case examples of cultural mistreatment compiled by our students.
2. *Self-Reflection & Management*: Explores concepts of bias, privilege, and microaggressions, as well as the value of Implicit Association Tests [24] for deepening self-awareness. Additional assignments allow participants to reflect on the relevance and application of these concepts using the Relationship Bridge (Fig. 1) and PAUSE model (Fig. 2; Table 1) within their work with trainees.
  **Fig. 1:**
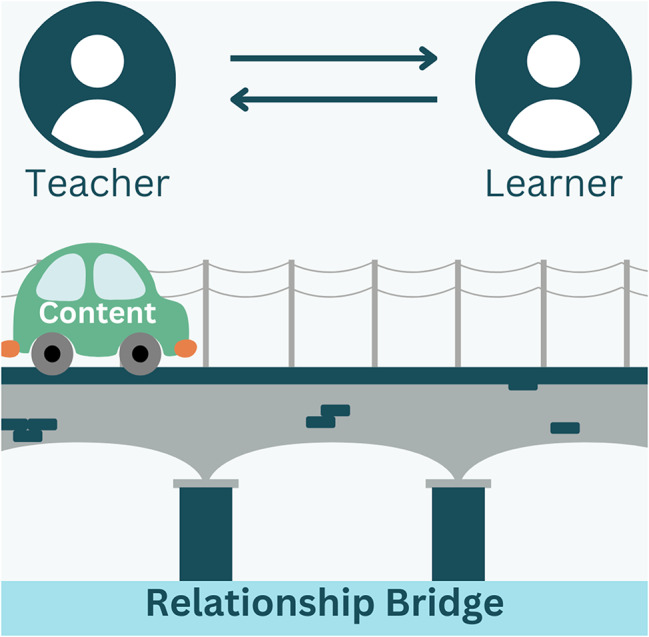
The Relationship Bridge
  **Table 1:** Core strategies and tools in SEED curriculum

Core Strategy	Details
1. Perspective-taking	Combines empathy as outlined by Brené Brown [25] with a reflection of data, narratives, and stories of harm as it has occurred in healthcare education.
2. The Relationship Bridge (Fig. 1)	Supports participants to explore the relationship between faculty and trainee and how it contributes to the learning experience for the trainees. In doing so, promotes dialogue among faculty that cultivates learning mediums that are meaningful for their students. [26]
3. The PAUSE Model (Fig. 2)	When harm has occurred, this model supports participants to self-reflect and manage their emotions before responding to the other party. This is an application of Emotional Intelligence and Diversity (EID), which encompasses the ability to feel, understand, articulate, manage, and apply the power of emotions to interactions across lines of difference. [27]
4. Triangulating Conflict [28] (Fig. 3)	Serves as a model to navigate challenging topics as empowered allies and increases comfort by providing difficult feedback critical for joint problem-solving. [29] It supports combining compromise with collaboration. [30] It also focuses on prioritizing healthier relations, mutual respect, and mutual purpose for an effective discussion of a conflict. [31]
5. An Effective Apology [32]	Emphasizes the value of acknowledging one’s role in the act of harm. It is critical to resolve conflict and support sustained relationships when unintentional harm occurs relating to a lack of awareness regarding inclusion and diversity issues.
  **Fig. 2:**
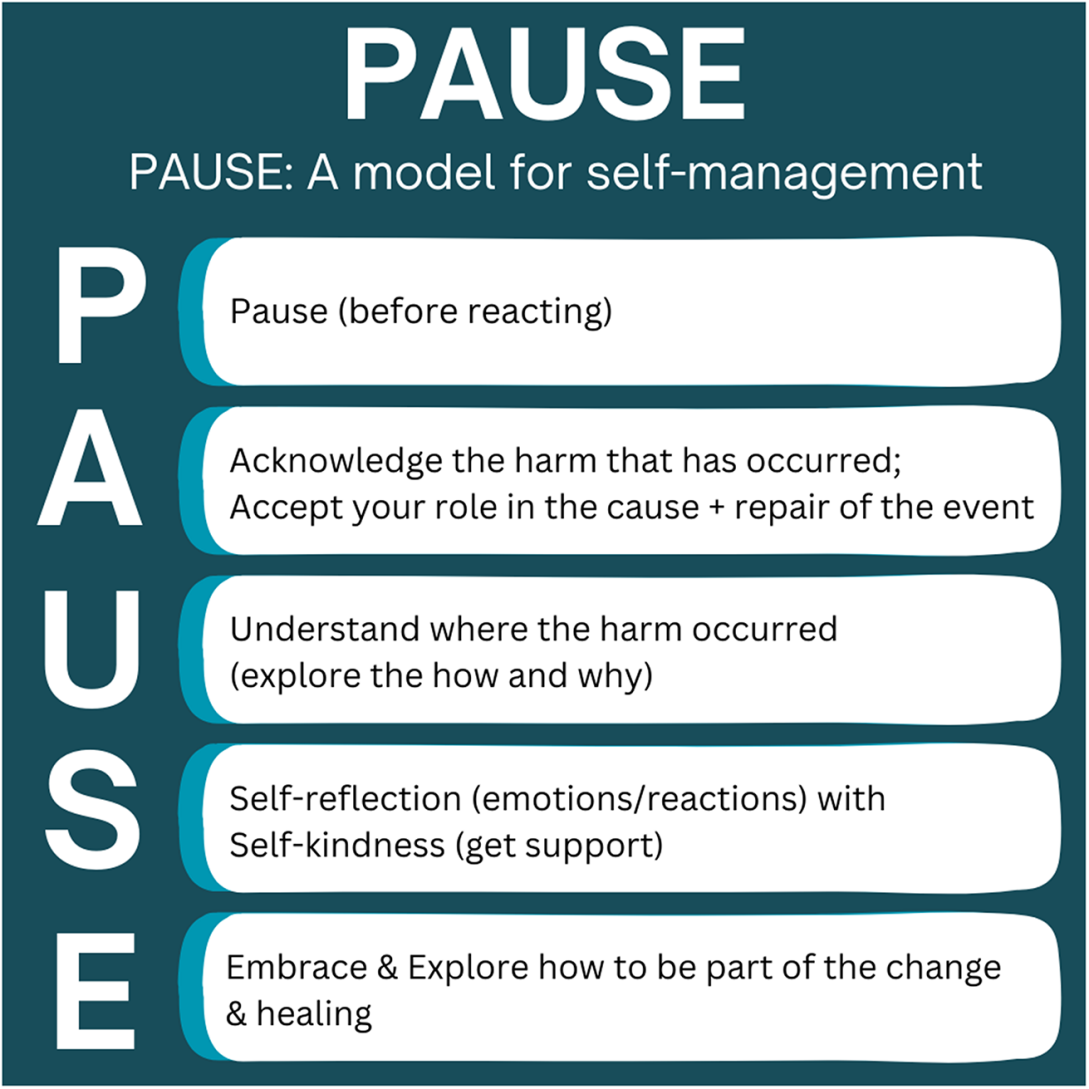
The PAUSE Model
  **Fig. 3:**
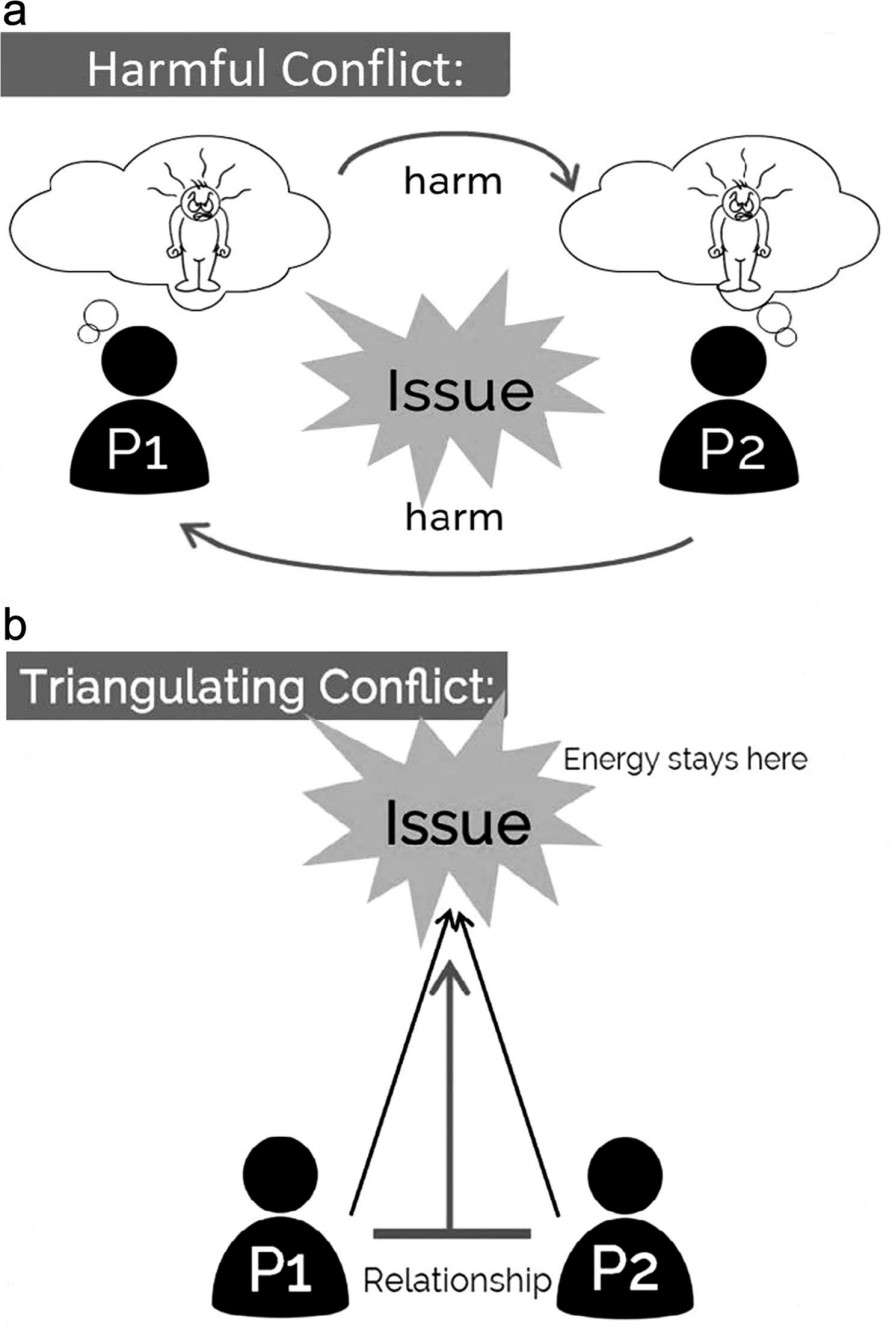
Triangulating Conflict [28]. (**a**) Harmful Conflict Dynamic. (**b**) Triangulation Method
3. *Inclusive Conversations*: This segment focuses on the application of core strategies to address cultural mistreatment with colleagues. These strategies (Table 1, items 1–3) are introduced through online learning, and with practical application through case discussion and role-plays in small groups.
4. *Diving Deeper with Consolidation*: Participants refine strategies learned in previous sections through roleplaying more complex situations, which include practice on how to effectively apologize when one contributes to cultural mistreatment and providing effective feedback to a colleague involved in cultural mistreatment. Then the session interaction shifts to enhance each participants’ strengths as allies and forming Allyship and ends the experience with *Let’s make it count*, a periodic self-reflection pause to empower each participant’s commitment to implementing course content and leaning into their work as effective educators for healthcare trainees. During in-person sections they were encouraged to discuss implementation strategies with their peers. (Table 1, items 4–5).

The University of California Davis Institutional Review Board reviewed this study and determined that it was “research not involving human subjects.” Data were collected from 6 cohorts beginning in September 2019 through February 2020. Pre-surveys were administered on registration for the course. Post-surveys were administered upon course completion and collected up to one week after in-person offering.

The pre and post-survey consisted of a total of 21 questions. There were 12 original questions asked across the full sample (*n* = 67 pre-test and *n* = 53 post-test) and 9 clinical questions added in later workshops (cohort 5 and 6) after incorporating clinical medical education. The pre and post-questionnaires consisted of Likert-type questions that asked respondents to indicate their agreement on a 5-point scale ranging from ‘strongly disagree’ (1) to ‘strongly agree’ (5). The questions focused on respondents’ awareness, learning, and practice of strategies related to culturally inclusive interactions. Statistical analysis involved the computation of summary statistics (means and standard deviations). Independent t-tests were used to determine if statistically significant differences existed between pre- and post-survey responses. Results at the *p* < .05 level were considered significant.

## Results

A total of 67 faculty participants completed the workshop including 20 educational leaders (Associate Deans, Associate Vice Chancellors, Course Instructors of Record, Department Chairs, and Directors). Of the 67 participants, 67 completed the pre-survey, and 53 completed the post-survey (100% and 79% response rate respectively). For clinical questions 1–9, 43 respondents completed the pre-survey, and 38 completed a post-survey. An independent pre-/post-test design was used to assess change in knowledge for this workshop. Participant-generated unique, identifiers were used to protect anonymity, but to allow for direct comparison of pre- and post-workshop knowledge.

Table 2 summarizes the results for all pre- and post-survey items. Results showed an overall increase in mean from pre-test to post-test. Results also indicated highly statistically significant (*p* < .001) differences from pre to post on all items, with one item statistically significant at a *p* < .05. The cumulative Cohen’s d for this sample is 1.55 showing a large effect size.

**Table 2:** Summary of self-perceived confidence in achieving tasks

	Pre-Test			Post-Test					
Item	*n*	Mean	SD	*N*	Mean	SD	95% CI for difference		2-tailed *p* value
Q1 - I can name at least 6 dimensions of culture in addition to race and ethnicity	67	3.06	1.205	53	4.23	0.776	1.527	0.807	*p* < .001
Q2- I can identify 3 elements of a culturally inclusive learning environment that positively contribute to teaching excellence.	67	3.03	1.058	53	4.11	0.8	1.419	0.747	*p* < .001
Q3 - I feel capable identifying uses of language, case examples, or imagery that perpetuate stereotypes, discrimination, or bias in the learning environment.	67	3.25	1.064	53	4.36	0.71	1.427	-0.783	*p* < .001
Q4 - I can define implicit bias.	67	3.49	1.021	53	4.45	0.722	1.276	0.645	*p* < .001
Q5- I am aware of how my biases impact my relationships with learners.	67	3.21	0.993	53	4.51	0.697	1.607	0.994	*p* < .001
Q6- I can define cultural privilege.	67	3.4	1.102	53	4.46	0.727	1.392	0.725	*p* < .001
Q7 - I can describe how cultural privilege can negatively impact the learning environment.	67	3.37	0.967	53	4.49	0.724	1.423	0.812	*p* < .001
Q8 - I have strategies for managing discomfort associated with receiving feedback related to issues of culture, bias and privilege.	67	3.12	1.008	53	4.11	0.64	1.294	0.694	*p* < .001
Q9 - I feel capable participating in difficult conversations related to culture, bias, or privilege.	67	3.06	1.043	53	4.04	0.733	1.300	0.656	*p* < .001
Q10 - I am capable of providing my faculty colleagues with appropriate feedback related to issues of culture, bias and privilege in learning environments.	67	2.94	1.071	53	3.85	0.818	1.25	0.567	*p* < .001
Q11- I can identify resources for helping faculty address issues of culture, bias and privilege in teaching.	67	2.96	0.991	53	3.96	0.733	1.319	0.695	*p* < .001
Q12- I can describe at least 3 methods for promoting safe environments for discussing topics such as race, bias, or privilege with students and colleagues	67	3.16	0.931	53	4.09	0.766	1.237	0.623	*p* < .001
C1 - I can identify 3 elements of a culturally inclusive clinical environment that positively contribute to clinical excellence.	43	3.14	0.99	38	4.00	0.697	1.236	0.485	*p* < .001
C2 - I feel capable identifying uses of language, case examples, or imagery that perpetuate stereotypes, discrimination, or bias in the clinical setting.	43	3.74	0.727	38	4.26	0.561	0.811	-0.215	0.001
C3 - I am aware of how my biases impact my relationships within clinical settings.	43	3.71	0.708	38	4.29	0.622	0.877	0.266	*p* < .001
C4 - I can describe how cultural privilege can negatively impact the clinical environment.	43	3.74	0.734	38	4.31	0.631	0.891	0.262	*p* < .001
C5 - I have strategies for managing discomfort associated with receiving feedback related to issues of culture, bias, and privilege in clinical settings.	43	3.12	0.889	38	4.06	0.583	1.272	0.601	*p* < .001
C6 - I can effectively participate in difficult conversations related to culture, bias, or privilege in clinical settings.	43	3.17	0.853	38	4.03	0.6	1.188	-0.533	*p* < .001
C7 - I can effectively provide my faculty colleagues with appropriate feedback related to issues of culture, bias and privilege in clinical settings.	43	2086	1.002	38	3.95	0.621	1.458	0.719	*p* < .001
C8 - I can identify resources for helping faculty address issues of culture, bias and privilege in clinical settings.	43	2.88	0.905	38	4.08	0.539	1.521	-0.869	*p* < .001
C9 - I can describe at least 3 methods for promoting safe environments for discussing topics such as race, bias, or privilege with trainees, patients and colleagues in clinical settings.	43	3.21	0.833	38	4.08	0.587	1.186	0.553	*p* < .001

Summary – independent t-test results for pre-post test

## Discussion

Our findings indicated there were positive changes in respondents’ learning and awareness with a large effect size. Available literature at the time of this study lacked quantitative data on the impact of curriculum efforts to address issues of culture and inclusion in healthcare learning environments [2, 4, 5, 33, 34]. These findings are being shared with the hope of supporting other medical schools and health institutions to improve the learning climate for students and healthcare environment at large [4]. This is a core part of healthcare community engagement.

Understanding the impact of power differences with self-awareness and humility in relationships is critical to addressing issues of privilege, bias, microaggressions, and stereotype threat. Future research could explore how cultural humility can account for various forms of entrenched inequity. Currently, SEED explores the concept of power differentials where we can support participants to better understand how structural inequity perpetuates these issues of harm. Self-awareness, self-assessment, and humility are core to navigating relationships to promote cultural inclusion and diversity. Designing educational activities that promote the practice of self-awareness, self-assessment skills, and humility in the context of these complex issues is critical [35]. 

Limitations of the study include trouble pairing more pre-and post-tests given they are anonymous and tracking is, therefore, more challenging. Greater effort will be made to pair scores for future cohorts of the SEED module. The study sample size is small and our participants are only from one institution. Longitudinal studies would help evaluate the long-term learning of participants. Including more qualitative research could add an understanding of how participants learned and applied the information. Self-reported increases in knowledge and skills may not translate to long-term behavioral changes.

Although this study was conducted among healthcare professionals, the professions included physicians, nurses, and staff of varied specialties and leadership levels. SEED has shifted to customized departmental, specialty-focused faculty development offerings as part of a systemic change effort sponsored by the Health System and School of Medicine’s Executive Leadership. To date, 678 individuals have been trained including 92% of School of Medicine and School of Nursing Leaders with over 50% of departments trained and additional 8 of 26 departments in the planning processes. SEED has been supportive at the institutional level and recognized by the Independent Student Analysis at the institution as a core support in meaningful change. SEED was also recognized as a core to ensuring that we met LCME (Liaison Committee on Medical Education) standards for mitigating mistreatment and promoting inclusion. Through this work, the SEED supports structural changes in subgroups of a healthcare system and empowers departments to lead change efforts. SEED expansion is also underway to resident trainees in a parallel learning process with an adapted curriculum for other staff in the healthcare education setting as well as interdisciplinary offerings. Intersectional frameworks of learning enhance the ability to support diverse populations [36]. Deeper evaluation including the development of longitudinal follow-up questionnaires is underway to further assess the longer-standing impact of the curriculum. Pilots have been offered for resident and fellow training in Psychiatry and Pediatrics Departments as well as Obstetrics and Gynecology. There is active exploration for staff offerings of the SEED curriculum. This curriculum has also been selected for the Faculty Education Research Award Grant of $100,000 through the American Board of Psychiatry and Neurology to pilot an asynchronous self-assessment offering as part of Maintenance of Certification for Board Certified Providers. Lessons learned include: (1) the importance of assessing the degree of harm that can occur through lack of inclusion, (2) the importance of Executive Leadership Sponsorship for efforts and buy-in, and (3) the value of collaboration with departmental leads to customize each offering for its participants.

## Conclusion

Our findings indicate that the SEED Faculty Development Curriculum show significant self-reported pre/post survey improvement when it comes to training medical educators on issues related to addressing issues of mistreatment, diversity, and inclusion. The success of the curriculum was derived from: (1) how materials were delivered, (2) the use of self-reflection, and (3) the strategic delivery of how educators can meaningfully apply SEED content to their work within healthcare. SEED is unique in providing vital yet practical communication tools and strategies to support cultural humility when navigating critical conversations related to diversity, inclusion, and harm. In addition, a collaborative partnership between faculty members and students allowed for optimal execution and buy-in from leadership and the institution to prioritize these conversations. In summary, the SEED Faculty Development Curriculum addressed mistreatment, bias, diversity, and inclusion within healthcare education with the effective application of novel tools to empower each participant as an active agent of change supporting healthcare trainees and the learning environment. Our findings suggest that SEED Curriculum has applicability across institutions at the local and national levels.

## Data Availability

Data is summarized within the manuscipt and raw data is provided within supplementary files.

## Abbreviations

SEED: Supporting Educational Excellence in Diversity
LGBTQ+: lesbian, gay, bisexual, transgender and queer acknowledging limitless gender identities and sexual orientations with “+”
AAMC: Association of American Medical Colleges
LCME: Liaison Committee on Medical Education
IRB: Institutional Review Board
